# Survey data of COVID-19 vaccine side effects among hospital staff in a national referral hospital in Indonesia

**DOI:** 10.1016/j.dib.2021.107098

**Published:** 2021-05-01

**Authors:** Dovy Djanas, Yusirwan, Rose Dinda Martini, Rahmadian, Hendria Putra, Adriani Zanir, Syahrial, Ricvan Dana Nindrea

**Affiliations:** a Department of Obstetrics and Gynecology, Dr. M Djamil General Hospital, Padang, Indonesia; b Department of Child Surgery, Dr. M Djamil General Hospital, Padang, Indonesia; c Department of Internal Medicine, Dr. M Djamil General Hospital, Padang, Indonesia; d Department of Finance, Dr. M Djamil General Hospital, Padang, Indonesia; e Department of Education and Research, Dr. M Djamil General Hospital, Padang, Indonesia; f Department of Nutrition, Faculty of Public Health, Universitas Andalas, Padang, Indonesia; g Department of Public Health and Community Medicine, Faculty of Medicine, Universitas Andalas, Padang, Indonesia

**Keywords:** COVID-19, Hospital, Side effects, Staff, Vaccine, Indonesia

## Abstract

In response to the current global challenge due to COVID-19, a dataset in this paper presented survey data of COVID-19 vaccine side effects among hospital staff in a national referral hospital in Indonesia. This survey data included the hospital staff of Dr. M. Djamil Hospital Padang, a national referral hospital in Indonesia, through a survey distributed via an online questionnaire, assessing COVID-19 vaccine side effects from 9th February to 13th February 2021. The items of the side effects included swelling, redness, itching, fever, headache, muscle pain, fatigue, coughing, diarrhea, nausea and vomiting, breathlessness, joint pain, fainted, anaphylactic reaction, itch, and swollen lymph nodes. In this survey data, we collected a total of 840 responses. The survey data were analyzed using univariate and bivariate analysis. Data analysis was performed using IBM version 25.0.

## Specifications Table

**Table:** 

Subject	Public health
Specific subject area	Health education, health promotion
Type of data	Primary data Tables Figure
How data were acquired	Data was collected using an online survey platform (Google Forms). The questionnaire is provided as a supplementary file.
Data format	Raw Analyzed
Parameters for data collection	The hospital staff who have received COVID-19 vaccine were identified through medical records review at Dr. M. Djamil General Hospital Padang, a national referral hospital in Indonesia. A total of 840 hospital staff were included to have survey assessing COVID-19 vaccine side effects, i.e., swelling, redness, itching, fever, headache, muscle pain, fatigue, coughing, diarrhea, nausea and vomiting, breathlessness, joint pain, fainted, anaphylactic reaction, itch, and swollen lymph nodes.
Description of data collection	The data was collected through an online questionnaire, which was sent to the hospital staff in a national referral hospital in Indonesia through convenience sampling technique.
Data source location	Region: Southeast Asia Country: Indonesia
Data accessibility	The dataset is provided as a supplementary file.

## Value of the Data

• The data is essential since this is the first survey involving large number of hospital staff in a national referral hospital in Indonesia to assess COVID-19 vaccine side effects, including swelling, redness, itching, fever, headache, muscle pain, fatigue, coughing, diarrhea, nausea and vomiting, breathlessness, joint pain, fainted, anaphylactic reaction, itch, and swollen lymph nodes.
• All researchers in communicable disease, epidemiology, and health promotion could benefit from these data since our findings can improve community knowledge and awareness about the COVID-19 vaccine, thus expected to increase public willingness to participate. Therefore, the data is necessary for health promotion and education to control COVID-19 transmission through vaccination and end the pandemic.
• The data is valuable to researchers who would like to compare our results with other studies on COVID-19 vaccine side effects from other countries, as well as to researchers who want to perform a systematic review and meta-analysis study in the future.
• These data may assist the government or health policymaker by providing scientific evidence-based data for developing COVID-19 related guidelines, as well as health policy formulations and implementation on COVID-19 vaccination.

## 1 Data Description

The dataset provides an insightful information based on survey data of COVID-19 vaccine side effects among hospital staff in a national referral hospital Indonesia, i.e., Dr. M. Djamil General Hospital Padang. The hospital staff who received COVID-19 vaccination were identified through medical records review. The survey data were collected from 840 hospital staffs in this national referral hospital to determine COVID-19 vaccine side effects, i.e., swelling, redness, itching, fever, headache, muscle pain, fatigue, coughing, diarrhea, nausea and vomiting, breathlessness, joint pain, fainted, anaphylactic reaction, itch, and swollen lymph nodes [1,2]. The questionnaire is provided as a supplementary file. The participant characteristics are presented in Table 1.

**Table 1:** Respondent characteristics (*n* = 840).

Characteristics	Category	f (%)
Sex	Male	270 (32.1)
	Female	570 (67.9)
Age (years)	< 20	3 (0.4)
	20–25	79 (9.4)
	26–30	205 (24.4)
	31–35	190 (22.6)
	36–40	120 (14.3)
	41–45	72 (8.6)
	46–50	70 (8.3)
	51–55	72 (8.6)
	56–60	26 (3.1)
	> 60	3 (0.4)
Professions	Midwife	29 (3.5)
	Nurse	363 (43.2)
	Medical doctor	115 (13.7)
	Medical specialist	49 (5.8)
	Non-medical staff	284 (33.8)
Educational background	Middle school	2 (0.2)
	High school	107 (12.7)
	Undergraduate degree	632 (75.2)
	Graduate degree	99 (11.8)
Living area	Downtown	594 (70.7)
	Outskirt	246 (29.3)
	Marriage	177 (88.5)

**Fig. 1:**
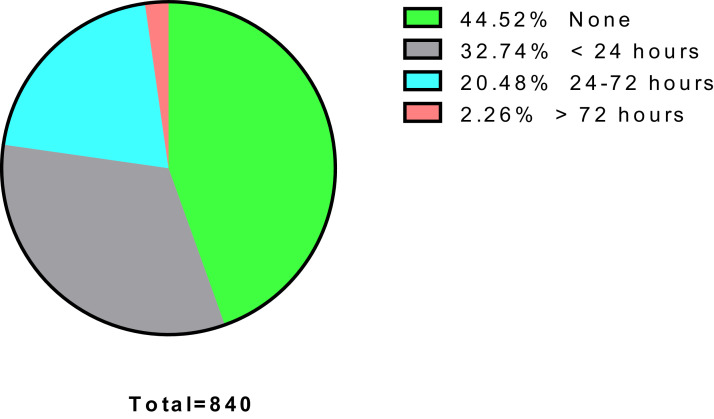
The symptoms time of COVID-19 side effects among hospital staff in a national referral hospital Indonesia.

The side effects data of COVID-19 vaccine among the hospital staff is presented in Table 2. The side effects of COVID-19 vaccine based on stratification according to medical staff and non-medical staff are presented in Table 3. The symptom onset of COVID-19 side effects among the hospital staff is presented in Fig. 1. The side effects of COVID-19 vaccine based on symptom onset among the hospital staff are described in Table 4. The association of side effects of COVID-19 vaccine with age of the hospital staff is presented in Table 5.

**Table 2:** The side effects of COVID-19 vaccine among hospital staff in a national referral hospital Indonesia.

Side Effects	Category	f (%)
Swelling	Yes	77 (9.2)
	No	763 (90.8)
Redness	Yes	17 (2.0)
	No	823 (98.0)
Itching	Yes	0
	No	840 (100.0)
Fever	Yes	13 (1.5)
	No	827 (98.5)
Headache	Yes	186 (22.1)
	No	654 (77.9)
Muscle pain	Yes	333 (39.6)
	No	507 (60.4)
Tiredness	Yes	301 (35.8)
	No	539 (64.2)
Coughing	Yes	66 (7.9)
	No	774 (92.1)
Diarrhea	Yes	26 (3.1)
	No	814 (96.9)
Nausea and vomiting	Yes	1.5
	No	98.5
Breathlessness	Yes	11 (1.3)
	No	829 (98.7)
Joint pain	Yes	13 (1.5)
	No	827 (98.5)
Fainted	Yes	1 (0.1)
	No	839 (99.9)
Anaphylactic reaction	Yes	3 (0.4)
	No	837 (99.6)
Tingling	Yes	58 (6.9)
	No	782 (93.1)
Swollen lymph nodes	Yes	4 (0.5)
	No	836 (99.5)

**Table 3:** The side effects of COVID-19 vaccine based on stratification according to medical staff and non-medical staff in a national referral hospital Indonesia.

	Hospital Staff	
	Medical Staff (*n* = 556)	Non-Medical Staff (*n* = 284)
Side Effects	(f/%)	(f/%)
Swelling	48 (8.6)	29 (10.2)
Redness	13 (2.3)	4 (1.4)
Itching	0	0
Fever	10 (1.8)	3 (1.1)
Headache	133 (23.9)	53 (18.7)
Muscle pain	233 (41.9)	100 (35.2)
Tiredness	201 (36.2)	100 (35.2)
Coughing	47 (8.5)	19 (6.7)
Diarrhea	20 (3.6)	6 (2.1)
Nausea and vomiting	11 (2.0)	2 (0.7)
Breathlessness	6 (1.1)	5 (1.8)
Joint pain	7 (1.3)	6 (2.1)
Fainted	0	1 (0.4)
Anaphylactic reaction	2 (0.4)	1 (0.4)
Tingling	37 (6.7)	21 (7.4)
Swollen lymph nodes	2 (0.4)	2 (0.7)

**Table 4:** The side effects of COVID-19 vaccine based on symptoms time among hospital staff in a national referral hospital Indonesia.

	Medical Staff (*n* = 335)		Non-Medical Staff (*n* = 131)	
Side Effects	< 24 h (*n* = 192) (f/%)	≥ 24 h (*n* = 143) (f/%)	< 24 h (*n* = 83) (f/%)	≥ 24 h (*n* = 48) (f/%)
Swelling	25 (13.0)	16 (11.2)	15 (18.1)	8 (16.7)
Redness	8 (4.2)	3 (2.1)	1 (1.2)	1 (2.1)
Itching	0	0	0	0
Fever	6 (3.1)	4 (2.8)	2 (2.4)	1 (2.1)
Headache	54 (28.1)	74 (51.7)	24 (28.9)	18 (37.5)
Muscle pain	114 (59.4)	91 (63.6)	39 (47.0)	31 (64.6)
Tiredness	81 (42.2)	94 (65.7)	48 (57.8)	28 (58.3)
Coughing	17 (8.9)	28 (19.6)	6 (7.2)	8 (16.7)
Diarrhea	5 (2.6)	15 (10.5)	2 (2.4)	4 (8.3)
Nausea and vomiting	7 (3.6)	4 (2.8)	0	1 (2.1)
Breathlessness	4 (2.1)	2 (1.4)	1 (1.2)	3 (6.3)
Joint pain	3 (1.6)	4 (2.8)	3 (3.6)	2 (4.2)
Fainted	0	0	0	0
Anaphylactic reaction	1 (0.5)	0	0	0
Tingling	15 (7.8)	21 (14.7)	9 (10.8)	8 (16.7)
Swollen lymph nodes	1 (0.5)	1 (0.7)	2 (2.4)	0

**Table 5:** The association of side effects of COVID-19 vaccine with age among hospital staff in a national referral hospital Indonesia.

	Age (*n* = 840)			
	>50 years (*n* = 101)	≤ 50 years (*n* = 739)		
Side Effects	(f/%)	(f/%)	p-value	OR (95% CI)
Swelling	5 (5.0)	72 (9.7)	0.167	0.48 (0.19–1.22)
Redness	1 (1.0)	16 (2.2)	0.709	0.45 (0.05–3.44)
Itching	0	0	N/A	N/A
Fever	2 (2.0)	11 (1.5)	0.663	1.33 (0.29–6.12)
Headache	19 (18.8)	167 (22.6)	0.464	0.79 (0.46–1.34)
Muscle pain	33 (32.7)	300 (40.6)	0.156	0.71 (0.45–1.10)
Tiredness	31 (30.7)	270 (36.5)	0.299	0.76 (0.49–1.20)
Coughing	7 (6.9)	59 (8.0)	0.864	0.85 (0.38–1.93)
Diarrhea	5 (5.0)	21 (2.8)	0.227	1.78 (0.65–4.83)
Nausea and vomiting	1 (1.0)	12 (1.6)	1.000	0.60 (0.07–4.70)
Breathlessness	3 (3.0)	8 (1.1)	0.136	2.79 (0.73–10.72)
Joint pain	1 (1.0)	12 (1.6)	1.000	0.60 (0.07–4.70)
Fainted	0	1 (0.1)	1.000	N/A
Anaphylactic reaction	0	3 (0.4)	1.000	N/A
Tingling	5 (5.0)	53 (7.2)	0.537	0.67 (0.26–1.72)
Swollen lymph nodes	1 (1.0)	3 (0.4)	0.402	2.45 (0.25–23.81)

N/A, not account; OR, odd ratio; *, significant at *p* < 0.05.

## 2 Experimental Design, Materials and Methods

This survey was performed using a cross-sectional method to determine COVID-19 side effects among hospital staff in a national referral hospital in Indonesia. This dataset was collected as a form of the commencement of the COVID-19 Sinovac vaccine vaccination in Indonesia which began on January 13rd, 2021. The vaccination was conducted in stages, in which the initial stage of vaccination was targeted at groups of hospital staff. Collection of datasets in Dr. M Djamil General Hospital Padang was performed after the vaccine administration to the medical and non-medical staff at Dr. M Djamil General Hospital Padang, gradually from January 18, 2021 to January 31, 2021. The questionnaire assessing the side effects of the COVID-19 vaccine was established based on infection prevention and control principles and procedures for COVID-19 vaccination activities in Indonesia [1]. Furthermore, the survey validation was performed by testing the survey before use. The survey validation showed good internal consistency for all items in the questionnaire with a Cronbach alpha value of 0.815. This dataset demonstrated the side effects of COVID-19 vaccination among medical and non-medical staff in one of the national referral hospitals in Indonesia, in which the majority of staff were at the group of age ≤ 50 years and the rest were at the group of age > 50 years. According to the International Council on Adult Immunization (ICAI), the latter mentioned group are more likely to have underlying comorbidities, and thus there is a greater need to prioritize this group in the vaccine distribution [2].

The dataset included 840 hospital staff in a national referral hospital in Indonesia identified through medical records review at Dr. M. Djamil General Hospital Padang, Indonesia. Written online informed consent was provided. The collection response data was conducted between February 9th and 13th, 2021. We preferred to use WhatsApp Messenger for enrolling potential participants. A questionnaire was presented in Google Forms and the link generated was then shared via WhatsApp Messenger after the contact number of participants was collected by medical records review with legal permission. The sampling technique in this dataset is convenience sampling [3]. The inclusion criteria included hospital staff who received COVID-19 vaccination with no comorbidity [4,5]. The univariate analysis was performed using frequency and percentage, while the bivariate analysis was conducted using the chi-square test. P-value of < 0.05 shows a statistically significant difference between groups. Odds ratios with 95% confidence interval was presented. All data analysis was performed using IBM version 25.0.

## Ethics Statement

This survey data passed the ethical review by the ethics commiittee of the Faculty of Medicine, Andalas University, Indonesia (No. 361/ KEP/ FK/ 2021). The survey data was conducted according to the Declaration of Helsinki.

## CRediT Author Statement

**Dovy Djanas:** Conceptualization, Investigation, Data curation, Writing – original draft; **Yusirwan:** Conceptualization, Methodology; **Rose Dinda Martini:** Conceptualization, Investigation; **Rahmadian:** Conceptualization, Investigation; **Hendria Putra:** Data curation, Investigation; **Adriani Zanir:** Data curation, Investigation, Methodolology; **Syahrial:** Investigation, Writing – original draft; **Ricvan Dana Nindrea:** Conceptualization, Formal analysis, Methodology, Visualization, Writing – original draft, Writing – review & editing.

## Declaration of Competing Interest

The authors declare that they have no known competing financial interests or personal relationships which have, or could be perceived to have, influenced the work reported in this article.

## Appendix Supplementary materials

Image, application 1

Image, application 2
