# Supplementary material for: Survey data of COVID-19 vaccine side effects among hospital staff in a national referral hospital in Indonesia
Source: Data Brief. 2021 May 1;36:107098. doi: 10.1016/j.dib.2021.107098 (PMC8087582; doi:10.1016/j.dib.2021.107098)
Supplement: Supplementary file 2 [file mmc2.doc]

**Questionnaire**

**Survey Data of COVID-19 Vaccine Side Effects among Hospital Staff in a National Referral Hospital Indonesia**

Assalamu Alaikum

I would like to thank you for taking your valuable time to complete our survey on **Survey Data of COVID-19 Vaccine Side Effects among Hospital Staff in a National Referral Hospital Indonesia**. The information you are about to provide is for research purposes only. The survey will take approximately 5 -10 minutes to finish. All of your responses will be kept strictly confidential and never disclosed your name. Your help is greatly appreciated.

If you have any questions about how to complete this questionnaire or if you have any additional comments or concerns you: would like to share regarding this survey, please feel free contact us.

Dr. Dovy Djanas

Dr. Ricvan Dana Nindrea

*Required

**Section A: Subject characteristics**

1. Sex*

Mark only one square

|  | Male |
| --- | --- |
|  | Female |

1. Age*

Mark only one square

|  | <20 years |
| --- | --- |
|  | ≥ 20-25 years |
|  | 26-30 years |
|  | 31-35 years |
|  | 36-40 years |
|  | 41-45 years |
|  | 46-50 years |
|  | 51-55 years |
|  | 56-60 years |
|  | > 60 years |

1. Professions*

Mark only one square

|  | Midwife |
| --- | --- |
|  | Nurse |
|  | Medical doctor |
|  | Medical specialist |
|  | Non-medical staf |

1. Educational background*

Mark only one square

|  | Junior high school |
| --- | --- |
|  | Senior high school |
|  | Bachelor degree |
|  | Master degree |

1. Living area*

Mark only one square

|  | Downtown |
| --- | --- |
|  | Outskirt |

**Section B: The side effects of COVID-19 vaccine**

1. Swelling*

Mark only one square

|  | Yes |
| --- | --- |
|  | No |

1. Redness*

Mark only one square

|  | Yes |
| --- | --- |
|  | No |

1. Itching*

Mark only one square

|  | Yes |
| --- | --- |
|  | No |

1. Fever*

Mark only one square

|  | Yes |
| --- | --- |
|  | No |

1. Headache*

Mark only one square

|  | Yes |
| --- | --- |
|  | No |

1. Muscle pain*

Mark only one square

|  | Yes |
| --- | --- |
|  | No |

1. Tiredness*

Mark only one square

|  | Yes |
| --- | --- |
|  | No |

1. Coughing*

Mark only one square

|  | Yes |
| --- | --- |
|  | No |

1. Diarrhea*

Mark only one square

|  | Yes |
| --- | --- |
|  | No |

1. Nausea and vomiting*

Mark only one square

|  | Yes |
| --- | --- |
|  | No |

1. Breathlessness*

Mark only one square

|  | Yes |
| --- | --- |
|  | No |

1. Joint pain*

Mark only one square

|  | Yes |
| --- | --- |
|  | No |

1. Fainted*

Mark only one square

|  | Yes |
| --- | --- |
|  | No |

1. Anaphylactic reaction*

Mark only one square

|  | Yes |
| --- | --- |
|  | No |

1. Tingling*

Mark only one square

|  | Yes |
| --- | --- |
|  | No |

1. Swollen lymph nodes*

Mark only one square

|  | Yes |
| --- | --- |
|  | No |

**Section C: Symptoms time**

1. The side effects of COVID-19 vaccine based on symptoms time*

Mark only one square

|  | None |
| --- | --- |
|  | < 24 hours |
|  | 24-72 hours |
|  | > 72 hours |
